# A Mobile App to Stabilize Daily Functional Activity of Breast Cancer Patients in Collaboration With the Physician: A Randomized Controlled Clinical Trial

**DOI:** 10.2196/jmir.6414

**Published:** 2016-09-06

**Authors:** Marco Egbring, Elmira Far, Malgorzata Roos, Michael Dietrich, Mathis Brauchbar, Gerd A Kullak-Ublick, Andreas Trojan

**Affiliations:** ^1^Department of Clinical Pharmacology and ToxicologyUniversity Hospital Zurich and University of ZurichZurichSwitzerland; ^2^EBPIDepartment of BiostatisticsUniversity of ZurichZurichSwitzerland; ^3^Department of Orthopedics and Emergency DepartmentWaid City HospitalZurichSwitzerland; ^4^Advocacy AGZurichSwitzerland; ^5^Breast-Center ZurichZurichSwitzerland

**Keywords:** collaboration, breast cancer, mobile app, daily functional activity, breast neoplasms

## Abstract

**Background:**

The well-being of breast cancer patients and reporting of adverse events require close monitoring. Mobile apps allow continuous recording of disease- and medication-related symptoms in patients undergoing chemotherapy.

**Objective:**

The aim of the study was to evaluate the effects of a mobile app on patient-reported daily functional activity in a supervised and unsupervised setting.

**Methods:**

We conducted a randomized controlled study of 139 breast cancer patients undergoing chemotherapy. Patient status was self-measured using Eastern Cooperative Oncology Group scoring and Common Terminology Criteria for Adverse Events. Participants were randomly assigned to a control group, an unsupervised group that used a mobile app to record data, or a supervised group that used the app and reviewed data with a physician. Primary outcome variables were change in daily functional activity and symptoms over three outpatient visits.

**Results:**

Functional activity scores declined in all groups from the first to second visit. However, from the second to third visit, only the supervised group improved, whereas the others continued to decline. Overall, the supervised group showed no significant difference from the first (median 90.85, IQR 30.67) to third visit (median 84.76, IQR 18.29, *P*=.72). Both app-using groups reported more distinct adverse events in the app than in the questionnaire (supervised: n=1033 vs n=656; unsupervised: n=852 vs n=823), although the unsupervised group reported more symptoms overall (n=4808) in the app than the supervised group (n=4463).

**Conclusions:**

The mobile app was associated with stabilized daily functional activity when used under collaborative review. App-using participants could more frequently report adverse events, and those under supervision made fewer and more precise entries than unsupervised participants. Our findings suggest that patient well-being and awareness of chemotherapy adverse effects can be improved by using a mobile app in collaboration with the treating physician.

**ClinicalTrial:**

ClinicalTrials.gov NCT02004496; https://clinicaltrials.gov/ct2/show/NCT02004496 (Archived by WebCite at http://www.webcitation.org/6k68FZHo2)

## Introduction

Telemedicine services have historically provided education, hardware, and software to patients and have been shown to improve functional status. If symptoms worsen, automatic algorithms to alert the nurse or physician have been demonstrated to be more effective than patient self-reporting alone [1]. The advent of mobile apps and the ease with which these are developed and used enables the stakeholders to collaborate in an increasingly dynamic and efficient manner.

Until now, mobile apps that allow continuous recording of disease- and medication-related symptoms have only occasionally been implemented in the management of patients undergoing chemotherapy [2]. However, symptom self-reporting on paper can only be explored with cumbersome efforts. In particular, therapeutic regimens in cancer patients require intensive monitoring procedures [3] because outpatient chemotherapy may frequently be accompanied by serious and potentially life-threatening adverse effects [4,5]. Well-informed patients spend significant efforts in documentation and management of symptoms as well as reviewing information related to both during the course of their disease and therapeutic interventions [6]. Consequently, involved physicians increasingly need to focus on collection and interpretation of a variety of information presented during the patient’s visit at first and after appropriate measures, if indicated.

Mobile apps have raised high expectations in various settings of daily life; their potential is now increasingly being examined in the health care sector. Supporting self-care with information on the disease or symptoms during routine cancer care can be enhanced by remote devices and patient collaboration through online patient groups and other forums [7]. In addition, recent studies have shown that the reporting of symptoms via email reminder and improved previsit preparation of physicians via printouts of symptoms may increase patients’ well-being [8]. Moreover, ancillary disease-related counseling with or without use of electronic devices successfully improved patient satisfaction through better understanding of specific symptoms, which in turn decreases patient anxiety [9]. Thus, available data indicate that mobile apps may well improve patient status if applied in a supervised setting in which the physician is empowered by additional information or services. However, the question remains whether self-quantifying apps, such as step counters, will provide added value simply by empowering the patient in an unsupervised setting.

Our objective was to explore the impact of a mobile and Web-based app in both an unsupervised setting and a setting supervised by the treating physician. We conducted a prospective randomized trial and invited early breast cancer patients to continuously record their symptoms according to the Common Terminology Criteria for Adverse Events (CTCAE) v4.0 via a novel electronic study device (mobile or Web app) and to also indicate their Eastern Cooperative Oncology Group (ECOG) Performance Status. Information for supportive care was also displayed by the app depending on the severity of symptoms upon data entry. The cumulative recorded data were made available to the patient in chart form (similar to a stock chart) at any time during the study. In the unsupervised setting, patients were instructed to use the app at any time except during study visits. In the supervised setting, the treating physician accessed the recorded data and patient-derived charts using the Web app during the study visit.

## Methods

### Study Design

We conducted a single center, three-arm, randomized, controlled, single-blinded interventional study. The protocol was approved by the Swiss Institutional Review Board (KEK-EK-ZH:2013-0200) and registered on ClinicalTrials.gov (NCT02004496). Patients with early breast cancer, aged 18 years and older, and initiating adjuvant or neoadjuvant chemotherapy at the Breast-Center Zürich were eligible to participate upon written informed consent. In addition, participants had to speak German and own a mobile phone.

### Study Groups

Eligible participants were recruited consecutively and without preselection during planning of chemotherapy. Participants received an envelope randomly assigning them to one of three study groups. The allocation sequence was concealed by the use of sealed, sequentially numbered, opaque envelopes. Group A (control) was the control group and received regular physician support. Group B (app) patients were instructed to use the mobile app without physician review. Group C (app and physician) patients used the mobile app and reviewed the reported data with the treating physician at scheduled visits. Participants in each group underwent three regular medical oncology visits scheduled on days 1, 21, and 42 during their chemotherapeutic intervention and independently of the study. The physician was single-blinded with regard to groups A and B. At the end of each visit, patients in all groups completed a questionnaire on paper. The observation period comprised 6 weeks and was initiated on the day of the first infusion therapy.

### Questionnaire

The questionnaire recorded performance status and daily functional activities according to the ECOG scale in an attempt to quantify daily levels of activity among breast cancer patients. The first five categories were presented as a visual analog scale; the sixth category, death, was omitted from the questionnaire. The ECOG activity score measure is routinely used to evaluate whether patients are eligible for chemotherapy, whether dose adjustments are required, and as an indicator for the need of palliative care. Additional questions were posed to further characterize the quality of care received by patients and the relationship between the patient and physician (Table 1). In addition, 30 preselected adverse events were listed with selectable severity, onset, and duration. The most frequent symptoms were selected according to clinical preview and experience; furthermore, patients could add additional symptoms as free text. Any medical measures undertaken could also be reported as free text. The questionnaire was issued to all groups at each of the three scheduled visits during the 6 weeks of study.

**Table 1:** Questions regarding the relationship between patient and physician for the three visits for each patient group.

Questions	Group and visit number, median (IQR)^a^								
	Control			App			App and physician		
	1	2	3	1	2	3	1	2	3
Concentration problems during visit	13 (69)	6^b^ (77)	17^b^ (64)	10 (51)	2^c^ (8)	4^c^ (10)	3 (29)	4^b,c^ (18)	4^c^ (12)
Well-informed about therapy	94 (15)	94 (35)	94 (20)	94 (20)	95 (20)	95 (14)	94 (21)	94 (14)	96 (12)
Well-informed about disease	92 (25)	94 (31)	94 (19)	92 (24)	93 (24)	95 (15)	96 (18)	96 (14)	96 (30)
Less likely to disfavor with care	4 (40)	6^b^ (73)	6 (17)	4 (10)	2^b,c^ (5)	4 (13)	2 (10)	1^c^ (8)	4 (6)
Awareness regarding adverse events	96 (23)	94 (14)	94 (15)	96 (21)	96 (18)	98 (13)	100 (13)	100 (14)	100 (15)
Satisfaction with medical care	96 (10)	96 (14)	95 (17)	99 (12)	96 (10)	98 (11)	100 (12)	99 (11)	100 (7)
Trust in data security	96 (14)	99 (13)	99 (13)	98 (13)	95 (11)	100 (8)	100 (11)	100 (7)	100 (8)
Feeling of being taken seriously	98 (18)	96 (17)	94 (19)	96 (17)	96 (11)	99 (11)	100 (12)	100 (8)	99 (8)

^a^ Value of 0 indicates complete disagreement, whereas 100 represents complete agreement with the statement in the question.

^b,c^ Annotations mark significant differences between the three groups. In this case three combinations of group pairs are possible. Two groups differ significantly in the answer to the question if they have different superscript characters.

### Mobile App

We developed a novel open-source mobile and Web app to record daily functional activity and adverse events. This mobile app was made available in the Apple and Google Android stores free of charge. Patients could report daily functional activity or symptoms with indication of severity in the electronic app device similar to the paper questionnaire. The visual analog scale from the questionnaire was substituted with a horizontal slider. Similar to the questionnaire, the label of symptom severity and category according to CTCAE was displayed below the slider. During the visits, nurses reminded the participants according to their randomization to use the app, but no other reminders were issued. Patients could edit a quick list of their preselected symptoms or select any of the 48 symptoms made available from the CTCAE listing. Only for group C was the treating physician enabled access to review and discuss the electronically reported symptoms during scheduled visits. Although the mobile app was publically available for download and use, only study patients who scanned an issued QR code containing a shared password could upload and store data securely. The development was frozen during the trial.

The variables of daily functional activity and severity of symptoms from the questionnaire and the mobile app were transformed to the same interval (0-100), if applicable. Missing data or withdrawn subjects were not excluded from the analysis to prevent selection bias for successfully compliant patients.

### Sample Size

We calculated the sample size for the three groups based on a 10% difference in daily functional activity. The significance level, after Bonferroni correction for the comparison of the three groups, was .016 (*P*=.05/3). A mean of 80% (SD 13%) was estimated by reviewing medical records of randomly selected patients at the Breast-Center Zürich. To detect differences with a power of 91%, a sample size of 50 patients per group was calculated. Given this sample size, a power of 80%, and a mean frequency of 4.4 (SD 3.3) adverse events, we expected to detect a difference in mean frequency of 2.2 adverse events per group. To minimize bias in the groups, only patients under the care of a single physician, who also participated as the study physician, were recruited.

### Statistical Analysis

All statistical tests were calculated using SPSS version 22.0 (IBM Corp, Armonk, NY, USA). Two-sided *P* values of less than .05 were considered to indicate statistical significance. For cases of more than two independent samples, Bonferroni correction was applied. Descriptive statistics, such as median and interquartile range, were computed. The Kruskal-Wallis test was used to identify differences in outcome variables among the three groups at all time intervals. The Mann-Whitney test further investigated differences between groups B and C. The paired Wilcoxon test analyzed the differences between time intervals within one group. Scatterplots and Spearman correlation (ρ) were used to validate the reported data from the app against the questionnaire.

## Results

### Baseline Characteristics

**Figure 1:**
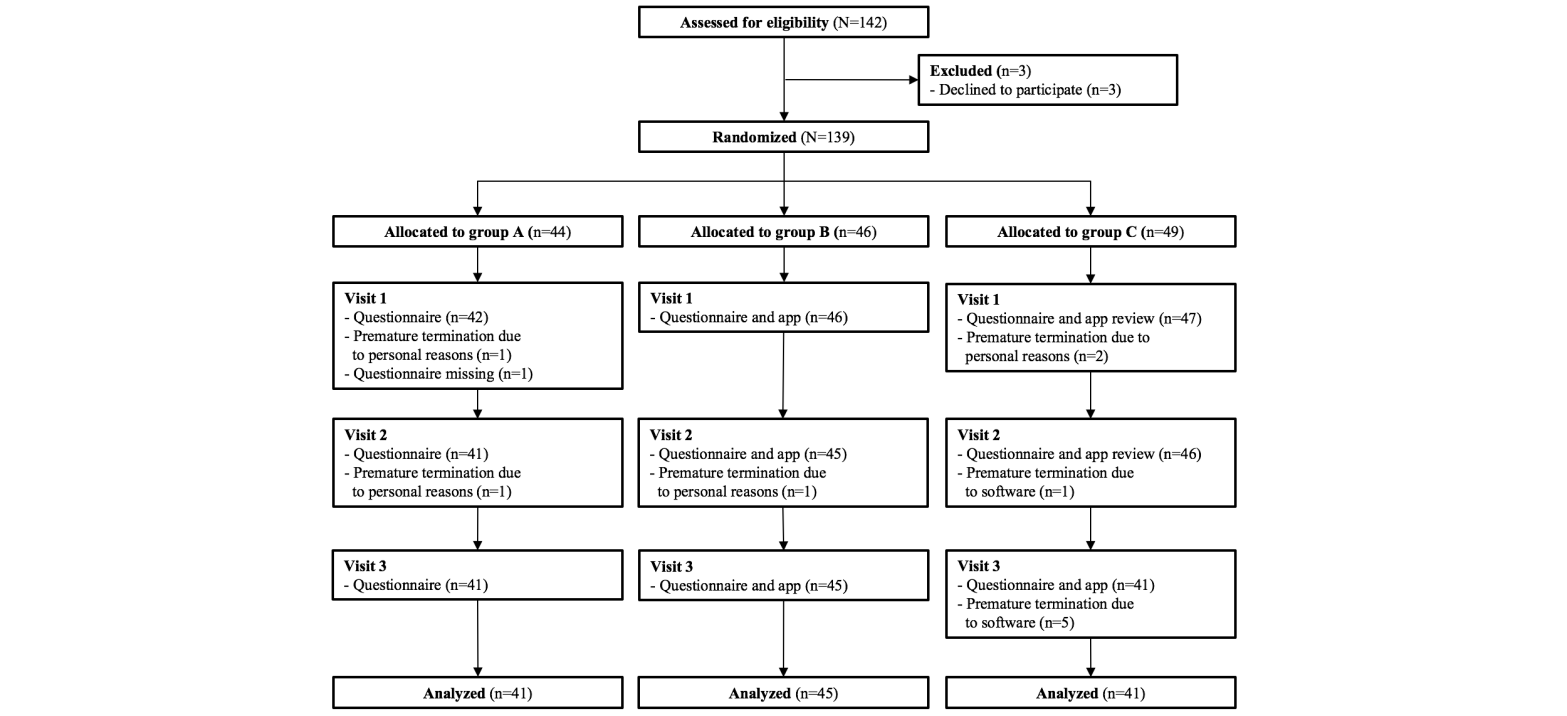
CONSORT diagram demonstrating the flow of patients.

Between December 2013 and July 2015, 139 patients were enrolled, 12 of whom did not complete the study for various reasons (Figure 1). Dislike of the constant confrontation with the disease was the reason for withdrawal for five of 12 patients who withdrew. The remaining 127 patients completed all three study visits. Baseline characteristics were equally distributed between the groups (Table 2). The most frequent chemotherapy regimen in all groups was epirubicin/cyclophosphamide (n=32), followed by paclitaxel/trastuzumab (control: n=8; app: n=4; app and physician: n=7), and paclitaxel/carboplatin (control: n=4; app: n=8; app and physician: n=7). In total, six different chemotherapy regimens were reported using seven distinct chemotherapeutic agents. The median observation interval between visit 1 and visit 2 (IQR 6), and also between visit 2 and visit 3 (IQR 8), was 21 days.

**Table 2:** Summary of baseline characteristics of participants.

Characteristics		All patients (N=139)	Control (n=44)	App (n=46)	App and physician (n=49)
Age (years), mean (SD)		53 (13)	56 (15)	50 (10)	53 (12)
Sex (female), n (%)		139 (100)	44 (100)	46 (100)	49 (100)
**Chemotherapy, n (%)**					
	Adjuvant	86 (61.9)	25 (57)	30 (65)	31 (63)
	Neoadjuvant	50 (36)	18 (41)	16 (35)	16 (33)
**Surgery, n (%)**					
	Biopsy (sentinel)	50 (36)	17 (39)	16 (35)	17 (35)
	Breast conserving	72 (51.8)	23 (52)	27 (59)	22 (45)
	Breast ablation	14 (10.1)	3 (7)	3 (7)	8 (16)
**Interval (days), median (IQR; range)**					
	Visits 1-2	21 (6; 6-43)	21 (6; 6-43)	20 (6; 7-27)	21 (2; 7-42)
	Visits 2-3	21 (8; 6-42)	21 (9; 7-42)	21 (7; 7-28)	20 (7; 6-28)
	Visits 1-3	42 (7; 13-84)	42 (2; 14-84)	41 (9; 26-49)	39 (7; 13-56)

### Daily Functional Activity

We collected 381 questionnaires from patients who completed the study. Current daily functional activity was indicated as the ECOG score at the time of the study visit, whereas the worst daily functional activity was defined as the worst rating before visits 1 to 3, respectively.

**Figure 2:**
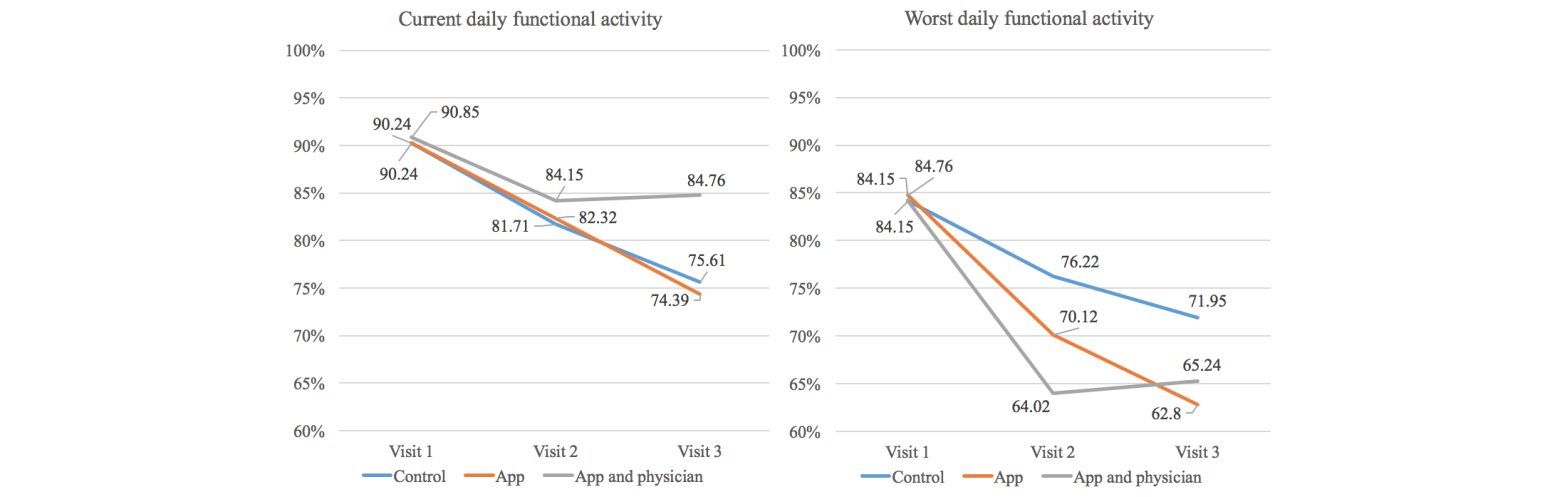
Visual analog scale for current and worst daily functional activity in the questionnaire.

As shown in Figure 2, both median current and worst daily functional activity declined in all patients during chemotherapy from the first to the second visit. From the second to the third visit, only patients in group C (app and physician) reported improvement in their functional activity, whereas scores in groups A (control) and B (app) continued to decline. From the first to the third visit, group A (current: median 90.24, IQR 19.63 vs median 75.61, IQR 21.95, *P*=.006; worst: median 84.15, IQR 23.93 vs median 71.95, IQR 32.32, *P*=.02) and group B (current: median 90.24, IQR 21.47 vs median 74.39, IQR 21.95, *P*=.02; worst: median 84.76, IQR 36.20 vs median 62.80, IQR 26.83, *P*<.001) showed a significant decline in functional activity in contrast to group C (current: median 90.85, IQR 30.67 vs median 84.76, IQR 18.29, *P*=.72; worst: median 84.15, IQR 39.88 vs median 65.24, IQR 32.32, *P*=.13). However, within the three groups differences in the reported functional activity at the three visits did not reach statistical significance, irrespective of whether current or worst daily functional activity was analyzed. Post hoc, the sample size for the actual standard deviation of 17% and a power of 91% should have required at least 83 patients per group in order to demonstrate a significant difference between the groups.

Overall, for groups B and C, results from the questionnaire were partially aligned with the data derived from app use. Patients started electronic recording of daily functional activity and symptoms in the app after the first visit, which also was the first day of treatment. The last data entry before each visit corresponded to the current daily functional activity indicated in the questionnaire at the visit. Similar to the results obtained from the questionnaire, the current daily functional activity (last value) declined significantly from the second (median 79.50, IQR 89.00) to the third visit (median 73.00, IQR 85.00) in group B (*P*=.007), but not in group C (median 75.00, IQR 90.00 vs median 70.00, IQR 84.00, *P*=.90). In addition, the best functional activity score of patients in group B (median 85.50, IQR 94.00 vs median 78.50, IQR 91.00, *P*=.008), but again not in group C (median 80.00, IQR 98.00 vs median 72.00, IQR 91.00, *P*=.34) dropped significantly in the first interval compared with the second interval.

In contrast, the median of the worst daily functional activity recorded in the app showed no significant difference in groups B and C between the first and second interval. The median over the total interval in the app-derived scores was not significantly lower for group B than for group C (median 45.50, IQR 49.00 vs median 45.00, IQR 70.00, *P*=.26) compared to almost identical results derived from the questionnaire for groups B and C (median 62.80, IQR 26.83 vs median 65.24, IQR 32.32, *P*=.07), respectively.

### Patient Communication

We further analyzed different aspects of the patient-physician relationship in each group (Table 1). In general, all patients felt very well informed about their disease and treatment, and were highly satisfied with their medical care. During the second visit, only group B reported significantly fewer concentration issues than group A (*P*=.006). In addition, group A was significantly less likely to express dissatisfaction with quality of care at the second visit than group C (*P*=.03). Most importantly, at the last visit, patients in groups B and C reported significantly fewer issues with concentration than patients in group A (*P*=.002).

### Patient Empowerment

**Figure 3:**
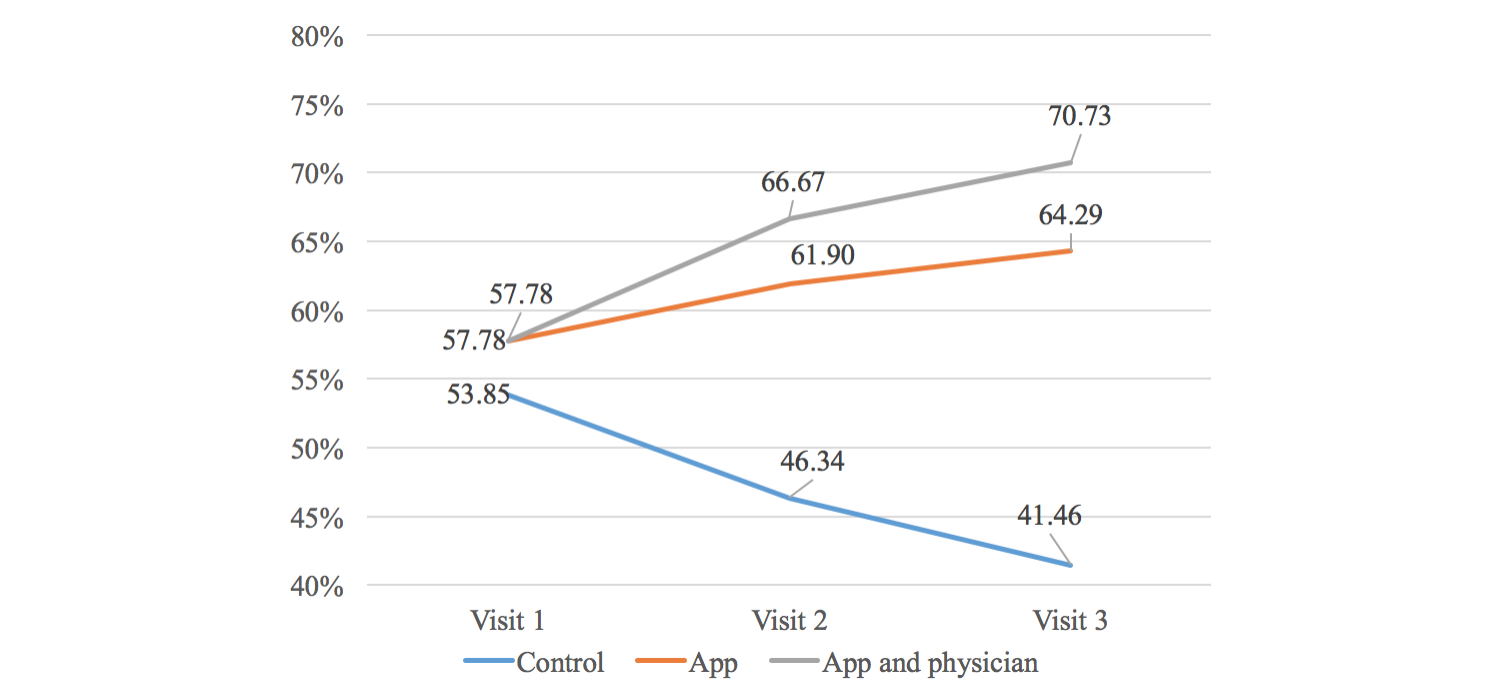
Participants who used the Internet to obtain disease-specific information.

As the study progressed, patients in all groups were significantly more likely to change their responses to the question about whether they used the Internet to obtain further information about their disease (Figure 3). At the third visit, significantly more patients in groups B (64.29% *P*=.04) and C (70.73%, *P*=.007) confirmed use of the Internet for this purpose compared with group A (41.46%).

### Symptom Reporting

The most frequently reported symptoms in the app were fatigue, hair loss, headache, and hypertension; from the questionnaire, fatigue, dry skin, headache, and sleep disorder were most prevalent. Regarding the number of distinct symptoms reported in the questionnaire and app, only group C showed a significant correlation in the interval from the first to the second visit (ρ=.381, *P*=.009) and from the second to the third visit (ρ=.362, *P*=.02), respectively.

Both groups reported more distinct symptoms in the app than in the questionnaire, a trend that was less prominent in group B than in group C. Therefore, the difference in numbers of distinct symptoms reported in the app versus the questionnaire was greater in group C (difference 377=1033−656) than in group B (difference 29=852−823). However, group B reported more symptoms in total (n=4808) in the app than group C (n=4463). Furthermore, group C reported significantly more distinct symptoms (median 13.00, IQR 12.00, *P*=.04) in the app for the total intervention interval, and more distinct mild symptoms (median 8.00, IQR 11.00, *P*=.02) in the interval from the first to the second visit compared with group B (distinct total: median 9.00, IQR 13.00; distinct mild: median 5.00, IQR 11.00). As for group A, the amount of distinct symptoms reported in the questionnaire was comparable to group C, whereas group B reported approximately 25% more symptoms.

## Discussion

### Principal Results

Few data exist that have addressed modalities and effects of electronic symptom reporting in patients undergoing chemotherapy. We demonstrate that a mobile app merits the potential to stabilize the daily functional activity of early breast cancer patients. Supervision and review of patient-reported symptoms in collaboration with their physician encouraged a timely and sincere discussion of symptoms reported in the app. Moreover, despite a rather short intervention period, supervised patients (group C) experienced a significant benefit with respect to daily functional activity in contrast to the unsupervised patients (group B and controls). These findings are in accordance with data gained from advanced cancer patients with longer follow-up managed by email prompts [8].

Patient-physician collaboration might have influenced the patients in three ways. First, the stabilization of daily functional activity seemed partially linked to a change in behavior, reflected by a more precise recording of symptoms in supervised patients, whereas fewer data entries were recorded than in unsupervised patients. Potentially augmenting existing mechanisms for symptom management along with routine oncology care, supervised app users also seemed to attain the ability to better differentiate and communicate their treatment-related symptoms, which consequentially facilitates appropriate management by the physician.

Furthermore, supervised patients were more likely than unsupervised patients and controls to communicate their dissatisfaction. It seems plausible that an increase in self-confidence may also positively affect daily functional activity. Strengthened self-confidence is likely reflected in decreasing incidence of concentration issues reported during the visits and an increasing use of the Internet to obtain further information about disease and related treatment in both groups using the mobile app. Previous studies also indicate that physicians, as well as the majority of patients, believe that mobile apps facilitate communication [10] and increase the frequency of discussion [11] during consultations.

In addition, patients receiving chemotherapy frequently report cognitive impairments [8]. In our study, both the supervised and unsupervised groups recorded improvements in worst daily functional activity in the app before the visit, although this became less evident from the questionnaires during the visit. The diary character of our mobile app is helpful for recalling disease-related information. This feature may have positive and negative consequences. Patients in group C reported impaired and probably more accurate scores for worst daily functional activity compared to the controls.

Six patients in group C withdrew from the study because of software reasons. One patient had functional problems with the recording of symptoms in the app. Five patients withdrew their participation from the study to avoid the constant confrontation with their disease. Of note, these patients in group C had the worst reported daily functional activity between the first and second visit. In general, group C was more likely to communicate their dissatisfaction with quality of care than group B and the controls. Maybe also patients in group B would have withdrawn from the study if their communication skills had been comparably strengthened. Furthermore, the daily recording of symptoms plus the supervision of symptoms during the visit in group C might be an additional burden. For some patients, this continuous workup of their symptoms might be too intense.

### Limitations

There are several limitations of this study. The three groups did not differ significantly from one another with respect to daily functional activity scores because the sample size turned out to be inadequate for the effect size observed. However, the three groups would have reported significantly different daily functional activity scores in favor of group C if withdrawn patients had not been included in the analysis. Similar findings have been reported for supervised cohorts of patients in previous studies [8,11].

Efforts were made to blind the physician to group A and B randomization. However, the physician may have inferred from the patients’ behavior whether they belonged to the control or the unsupervised app group. Unblinding of the physician and the patient to the intervention may have affected the patients’ responses to the questions, especially because the patient completed the questionnaire following consultation with the physician. However, patients reported congruent answers in the mobile app outside the clinic.

Different chemotherapeutic regimens may cause different adverse events. We cannot exclude that effects observed on daily functional activity were thereby affected. We maintain, however, that the randomized design of the study and a balanced distribution of treatment regimens renders a systematic bias unlikely.

It should be noted that no alert signals about technical issues and data safety were raised during the entire course of the study.

### Conclusion

Mobile apps increasingly contribute to patient education, disease self-management, and remote monitoring of patients [12]. We demonstrate that only a collaborative review of timely reported and naïve, although patient-derived, symptoms has beneficial effects on daily living activity in early breast cancer patients. The use of mobile apps under supervision may enable patients to report adverse events more precisely in the context of increasingly complex cancer therapies and limited resources.

## Abbreviations

CTCAE: Common Terminology Criteria for Adverse Events
ECOG: Eastern Cooperative Oncology Group

## Multimedia Appendix 1

CONSORT EHEALTH Checklist (V 1.6.1).
